# A Novel Fully Automated Deep Learning Model for Coronary Artery Calcification Detection on Computed Tomography

**DOI:** 10.3390/diagnostics16050646

**Published:** 2026-02-24

**Authors:** Turki Nasser Alnasser, Alireza Hokmabadi, Michael J. Sharkey, Ahmed Maiter, Krit Dwivedi, Mahan Salehi, Christopher Johns, Smitha Rajaram, Kavitasagary Karunasaagarar, David G. Kiely, Samer Alabed, Andrew J. Swift

**Affiliations:** 1 School of Medicine & Population Health, The University of Sheffield, Sheffield S10 2TN, UKa.j.swift@sheffield.ac.uk (A.J.S.); 2 Radiological Sciences Department, College of Applied Medical Science, King Saud bin Abdulaziz University for Health Science, Riyadh 11426, Saudi Arabia; 3 King Abdullah International Medical Research Center (KAIMRC), Riyadh 11426, Saudi Arabia; 4 Insigneo Institute, Faculty of Engineering, The University of Sheffield, Sheffield S10 2TN, UK; 5 3D Imaging Lab, Sheffield Teaching Hospitals NHS Foundation Trust, Sheffield S10 2TN, UK; 6 Department of Clinical Radiology, Sheffield Teaching Hospitals, Sheffield S10 2TN, UK; 7 National Institute for Health and Care Research, Sheffield Biomedical Research Centre, Sheffield S10 2TN, UK; 8 Sheffield Pulmonary Vascular Disease Unit, Sheffield Teaching Hospitals NHS Foundation Trust, Sheffield S10 2TN, UK

**Keywords:** non-contrast CT, coronary, calcifications, pulmonary hypertension

## Abstract

**Objectives**: To assess the diagnostic accuracy of a fully automated deep learning (DL) model for coronary artery segmentation and calcification detection on non-contrast, non-gated CT scans. **Methods**: A two-stage 3D segmentation pipeline was developed using 42 non-contrast and 27 contrast-enhanced CT scans to identify coronary artery calcifications in the right coronary artery (RCA), left anterior descending artery (LAD), and left circumflex artery (LCX). The model was trained with anatomically refined labels and region-based optimisation to improve structural coherence. Model outputs were visually assessed in a separate cohort of 100 scans by two independent, experienced observers. Segmentation and detection performance were evaluated against manually annotated reference standards using a binary analysis in 473 internal and external scans. Volumetric measurements of calcifications were compared with baseline severity gradings derived from radiologist reports. **Results**: Most model outputs were rated as excellent in the visual assessment, with good agreement between the outputs and manual reference standards for coronary artery segmentation (κ 0.68 to 0.81) and calcification detection (κ 0.79 to 0.85). The model accurately detected the presence of calcifications in the RCA (κ = 0.82, *p* < 0.001), LAD (κ = 0.93, *p* < 0.001), and LCX (κ = 0.82, *p* < 0.001). The diagnostic accuracy metrics of the model for calcification detection were: sensitivity, 95%; specificity, 98%; positive predictive value, 99%; and negative predictive value, 88%. The volume of calcification yielded by the model correlated with radiologist-reported disease severity, with regression coefficients of 28.3 for RCA, 28.7 for LAD, and 77.5 for LCX. **Conclusions**: The developed DL model segmented the coronary arteries, detected the presence of calcifications, and predicted disease severity with high accuracy.

## 1. Introduction

Coronary artery disease (CAD) is a leading cause of mortality from cardiovascular disease. The degree of mural calcification correlates closely with the burden of atherosclerotic plaque within the coronary arteries, and therefore the degree of stenosis and severity of disease [1,2,3,4,5,6]. The ability to detect and quantify the burden and extent of coronary artery calcifications is important for diagnosis, prognostication and treatment planning of CAD [1,2,3]. While coronary calcification was initially recognised on fluoroscopy in the 1960s, the first standardised method for its quantification was established by Agatston and Janowitz in the 1990s. Coronary artery calcium (CAC) scoring is usually performed using electrocardiographically (ECG) gated cardiac computed tomography (CT) scans as the reference standard by calculating the calcium area in square millimetres and density in Hounsfield units (HU) [7,8].

Non-contrast, non-gated CT of the thorax is commonly performed for a variety of indications, including evaluation of interstitial lung disease, lung cancer screening and monitoring, in patients with allergy to iodinated contrast and CAC scoring. Several professional societies, including the Society of Cardiovascular Computed Tomography (SCCT), the Society of Thoracic Radiology (STR), and the American College of Radiology (ACR), strongly recommend that CAC be assessed and not overlooked on all non-dedicated CT scans [8,9,10]. Over 7 million non-contrast CT scans of the thorax are performed annually, which could be used for CAC assessment and early detection of CAD [11]. In addition, it has been demonstrated that non-ECG-gated non-contrast CT can provide accurate and reproducible CAC scoring comparable to ECG-gated scans [8,11].

Manual segmentation of cardiothoracic structures is often time-consuming, highly subjective, and associated with high costs, which can result in variability between operators and inconsistencies in the delineation of anatomical structures, particularly in complex or small regions [12,13,14,15,16,17]. For coronary arteries, these limitations are even more pronounced due to their small size. These challenges underscore the need for automated segmentation methods, which could help reduce costs, save time, and minimise errors [15,16,17].

Artificial intelligence (AI) tools have demonstrated the ability to perform accurate segmentation and quantitative measurement of thoracic structures on CT [12,13,14]. Deep learning (DL) models for segmentation of the coronary arteries have shown potential to improve the accuracy, reliability and efficiency of evaluating CAC [15,16]. For example, previous work has used a 3D Dense-U-Net to segment the coronary arteries from CT angiography scans. The model performance was assessed using the Dice Similarity Coefficient (DSC) and Intersection over Union (IoU). It achieved high accuracy, with a DSC greater than 0.95 and an IoU greater than 0.94, performing at least as well as a standard clinical tool on major coronary arteries while providing better detection of smaller vessel branches. The model processed each scan in approximately 10–15 s [17]. Another study employed a convolutional neural network (CNN)-based model for automatic coronary artery segmentation and quantification. The model was developed to detect and measure CAC from both dedicated ECG-gated cardiac CT and routine non-gated chest CT scans. The automated approach reduced analysis time, processing scans in approximately 3.5 s compared with 261 s for manual assessment. It reported sensitivity values of 71–94% and positive predictive values (PPV) of 88–100% in comparison to the reference standard [15].

While the current literature has focused primarily on ECG-gated CT scans for CAC scoring, this study aimed to develop a fully automated DL model for the accurate segmentation of the coronary arteries and detection of calcifications in patients with different forms of pulmonary hypertension (PH), using non-ECG-gated CT scans, including both contrast-enhanced and non-contrast acquisitions, and focused on segmenting the major coronary arteries and quantifying their volumes. Its diagnostic accuracy was assessed using routine non-contrast, non-ECG-gated CT scans from internal and external cohorts from seven hospitals across England.

## 2. Materials and Methods

### 2.1. Ethics Approval

The study was conducted in accordance with the Declaration of Helsinki and approved by the Institutional Review Board (Ethic Committee Name: East of England-Cambridge East Research Ethics C, Approval Code: REC 22/EE/0011, Approval Date: 24 February 2022). The Assessing the Spectrum of Pulmonary Hypertension Identified at a Referral Centre (ASPIRE) registry is an ethically approved research database managed by Sheffield Teaching Hospitals NHS Foundation Trust (Reference: STH14169, REC 22/EE/0011). In accordance with UK regulations, written consent was not required for analysis of routinely collected de-identified clinical data collated as part of an ethically approved research database (Reference: STH14169, REC 22/EE/0011). (National guidance within IRAS: https://www.myresearchproject.org.uk/help/hlpcollatedqsg-nhsrec.aspx#1421, accessed on 15 January 2026).

### 2.2. Study Population and Included CT Scans

Eligible patients and their CT scans were identified retrospectively from the Assessing the Spectrum of Pulmonary Hypertension Identified at a Referral Centre (ASPIRE) registry. The registry comprises consecutive patients who have undergone investigation for suspected PH at the Sheffield Pulmonary Vascular Disease Unit and had not received any prior therapy. Patients within the registry have typically undergone specialist clinical review, lung function testing, multimodal imaging and right heart catheterisation (RHC) as previously described [18]. Patients were excluded if they had received treatment for PH before specialist referral, or if PH was attributable to multiple unrelated causes. In this study, adult patients ≥ 18 years within the registry were eligible for inclusion if they had undergone non-contrast CT and were being investigated for shortness of breath and/or PH. CT scans were excluded if they comprised fewer than 100 slices in order to preserve segmentation quality across multiple coronary artery slices. All CT scans were acquired using multidetector CT scanners with standard acquisition helical mode parameters: pitch 1, tube current 100–500 milliampere, 120 kilovolts, a field-of-view of 400 × 400 mm and an acquisition matrix of 512 × 512.

### 2.3. Study Flow and Data Splitting

**Figure 1:**
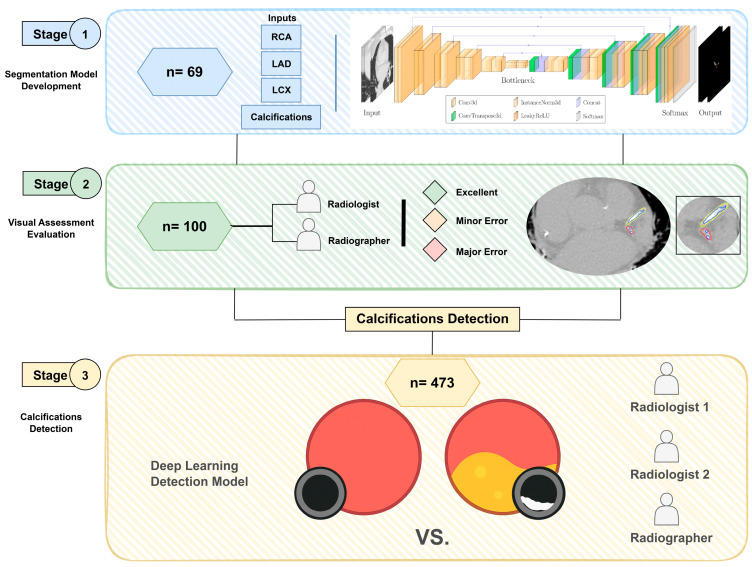
Flow chart of the study design, including (1) coronary segmentation model development stage, (2) visual assessment of model outputs stage and (3) calcifications detection stage.

The flow of the study is summarised in Figure 1. In Stage 1, a deep learning model for coronary artery segmentation and calcification detection was trained and tested using 69 CT scans (42 non-contrast and 27 contrast-enhanced). In Stage 2, the model outputs were assessed visually using 100 non-contrast CT scans. In Stage 3, the accuracy of the model for the detection of calcification was tested using 473 internal and external non-contrast CT scans.

### 2.4. Stage 1: Model Development

#### 2.4.1. Data Preparation

The segmentation model was developed using a dataset of 69 CT scans (42 non-contrast and 27 contrast-enhanced). Given the relatively small number of cases, 85% (56/69) of scans were allocated for training, with the remaining 15% (13/69) used for testing. To ensure robustness and assess performance variability, a 5-fold cross-validation was employed on the training set, while extensive data augmentation was applied to mitigate the effects of limited sample size.

**Figure 2:**
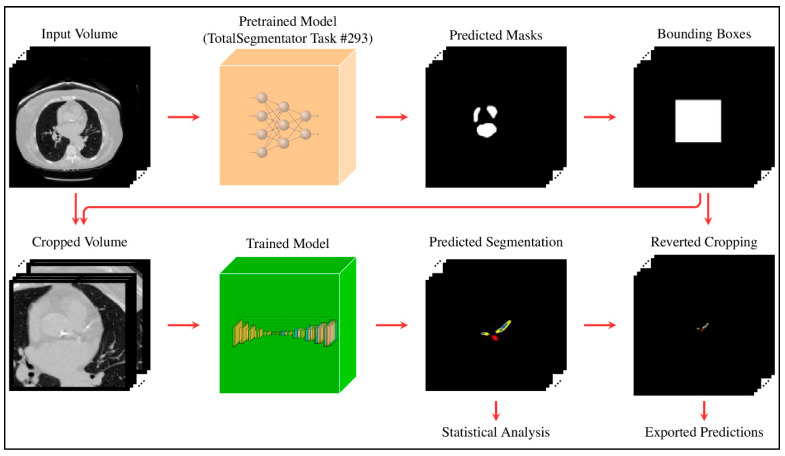
Two-stage pipeline: initial heart segmentation is used to crop the region of interest; predictions are then made on the cropped volume and mapped back to the original space.

All images were resampled to a voxel spacing of (0.8, 0.73, 0.73) mm to ensure consistency across the dataset. To focus the model on the region of interest and reduce computational overhead, a two-stage cascade pipeline was implemented. In the first stage, pretrained weights from TotalSegmentator [19] (sub-task ID 293 of the “total” task) were used to segment the heart. Based on this output, the heart region was extracted by cropping around the heart label with a padding of (10, 10, 0). For each subject, the original and cropped image shapes, along with the bounding box coordinates, were saved to enable reconstruction of the full-size prediction at the end of the second stage. This procedure was applied consistently to both the training and test sets. The full pipeline is illustrated in Figure 2.

#### 2.4.2. Reference Standard

The reference standard annotations were created by a cardiothoracic radiologist (AJS) using MASS software (Version 2021EXP, Leiden University Medical Center, Leiden, The Netherlands). The labels were originally provided as .con files, which were first decoded and converted to NIfTI format, consistent with the image data, to make them compatible with the model input.

The initial annotations included four classes: right coronary artery (RCA), mainstem and left anterior descending coronary artery (LAD), left circumflex coronary artery (LCX), and a single annotation for calcifications associated with any artery. To improve anatomical specificity, binary logical operations (e.g., intersection and subtraction between vessel and calcification masks) were applied to isolate calcifications associated with each artery, resulting in six distinct classes: RCA, mainstem + LAD, LCX, and their corresponding calcifications.

The 3D full-resolution nnU-Net configuration [20] was used as the second-stage model. Although nnU-Net provides a robust baseline for medical image segmentation, achieving optimal performance still requires careful dataset preparation, thoughtful label engineering, and tuning of training parameters. We experimented with multiple label configurations and found that region-based training [21], in which composite regions are defined as unions of related labels, significantly improved performance. Specifically, each coronary artery was grouped with its associated calcification to form three regions: whole_RCA (RCA + calcification), whole_LAD (LAD + calcification), and whole_LCX (LCX + calcification), while still retaining the three individual calcification labels. This configuration enabled the model to learn more anatomically coherent structures while predicting six distinct labels. We found that this approach substantially outperformed training with individual labels alone or without region merging.

#### 2.4.3. Model Architecture and Training

The network architecture consisted of five encoding and decoding layers plus a bottleneck, with each level comprising two cascaded 3D convolutional layers, followed by instance normalisation and LeakyReLU activation. Full architectural details are provided in Supplemental Table S1.

The model was trained for 250 epochs using a batch size of 2 and an input patch size of 112 × 128 × 160. To enhance generalisation and compensate for the limited dataset size, nnU-Net’s built-in data augmentation pipeline was used, which includes affine transformations, Gaussian noise, axis flipping, and elastic deformations. Training was guided by a combined loss function consisting of Dice similarity coefficient (DSC) and cross-entropy losses with equal weighting, allowing effective optimisation of both class overlap and voxel-wise accuracy. Although our dataset did not suffer from severe class imbalance, adaptive patch sampling still improves the representation of smaller or less frequent structures. Final predictions were obtained by ensembling the five cross-validated models.

#### 2.4.4. Experiment Setup

All experiments were conducted on a laboratory server equipped with NVIDIA H100 GPUs (96 GB memory), running Linux with Python 3.11, PyTorch 2.5.1, and CUDA 12.4. Training was performed using a single GPU per fold in parallel across the available GPUs, with a total training time of approximately 6 h per fold. Although high-performance hardware was used to accelerate cross-validation, the proposed model has a modest memory footprint and can be trained on substantially lower-end GPUs (e.g., ~10 GB VRAM), with increased training time.

#### 2.4.5. Ablation Study

To assess the contribution of key components of the proposed pipeline, an ablation study was performed by systematically removing heart cropping and region-based training. Three models were compared: (i) the full proposed model, (ii) a model trained without heart cropping, and (iii) a model trained without region-based label merging. All models were evaluated using binary detection of coronary artery calcification (presence vs. absence) against the manual reference standard in the independent cohort (*n* = 473).

The results presented in Supplementary Table S2 demonstrate that heart cropping improved specificity and reduced false-positive detections, while region-based training improved performance, particularly in smaller vessels (RCA and LCX). The full model showed the most balanced and consistent performance across all vessels and for overall calcification presence.

### 2.5. Stage 2: Visual Assessment of Model Outputs

A cohort of 100 non-contrast CT scans was assessed visually for segmentation evaluation, rather than reporting DSC and Hausdorff Distance, due to the small size of the anatomical structures, which makes employing region-based or surface distance indicators challenging. The visual assessment was conducted independently by a cardiothoracic radiologist with 13 years of experience (AJS) and a cross-sectional imaging radiographer with five years of experience (TNA) based on a quality assessment scale in which ‘excellent’ represents highly reliable segmentation, ‘minor error’ reflects errors that do not influence segmentation, and ‘major error’ denotes errors perceived by the rater as affecting the segmentation (Supplemental Table S3). The observers were blinded to the clinical and imaging data, as well as to each other’s assessments. Inter-observer agreement of visual assessment scores was assessed using free-marginal Kappa (κ) analysis, which accounts for situations where raters are free to assign any number of items to each category, without assuming a fixed distribution of ratings [22].

### 2.6. Stage 3: Diagnostic Accuracy of Calcification Detection

The diagnostic accuracy of the model for the binary detection of coronary artery calcification was tested using 473 non-contrast CT scans. Expert opinion from a cardiothoracic radiologist (AJS) and a radiographer (TNA) for the presence and severity of coronary artery calcifications was used as the reference standard. Sensitivity, specificity, positive predictive value (PPV), negative predictive value (NPV), and the area under the receiver operating characteristic curve (AUC) were calculated from 2 × 2 contingency tables. Agreement between the model result and the expert radiologist’s opinion was measured using Cohen’s κ, with the strength of agreement interpreted according to [23].

Ordinal regression analysis was conducted to assess the association between DL-derived coronary calcification volume and radiologist-assigned severity scores for each coronary artery (i.e., RCA, LAD, and LCX). Radiologist severity scores were treated as ordinal outcome variables (1 = mild, 2 = moderate, 3 = severe), and calcification volumes (measured in mm^3^) were used as continuous predictors. The regression coefficient (B), 95% confidence interval (CI), Wald statistic, and *p*-value were reported for each artery.

### 2.7. Statistical Analysis

All statistical analyses in Stage 2 and Stage 3 were performed using SPSS Statistics for Windows (Version 29.0.IBM Corp, 2022, Armonk, NY, USA). The statistical significance threshold α was defined as 0.05.

## 3. Results

### 3.1. Patient Characteristics

The demographics of the included patients are summarised in Table 1. Stage 1 cohort includes 36 males and 33 females with an average age of 69 ± 13 years. The cohort comprises eight patients with pulmonary arterial hypertension (PAH), four with left heart disease, 21 with lung diseases, three with chronic thromboembolic pulmonary hypertension (CTEPH), six without PH and 27 with suspected pulmonary embolism (PE). Stage 2 cohort includes 33 males and 67 females with an average age of 66 ± 12 years. The cohort includes 33 patients with PAH, 17 with left heart disease, 14 with lung diseases, seven with CTEPH, and 29 without PH. The Stage 3 cohort includes 175 males and 298 females with an average age of 64 ± 13 years. In this cohort, 80% of patients were scanned at Sheffield Hospital, while the remaining 20% were scanned across seven other hospitals throughout England. The cohort consisted of 159 patients with PAH, 72 with left heart disease, 89 with lung diseases, 38 with CTEPH, and six with unclear PH. Of those, 109 patients were diagnosed as PH-negative (23%). Among the three cohorts, calcifications were present in 51% of cases within the RCA, 72% within the LAD, and 46% within the LCX. The patients were scanned using different scanners, including GE HealthCare (72%), Canon (23%), and Siemens (5%).

### 3.2. Stage 2: Visual Assessment of Model Outputs

**Figure 3:**
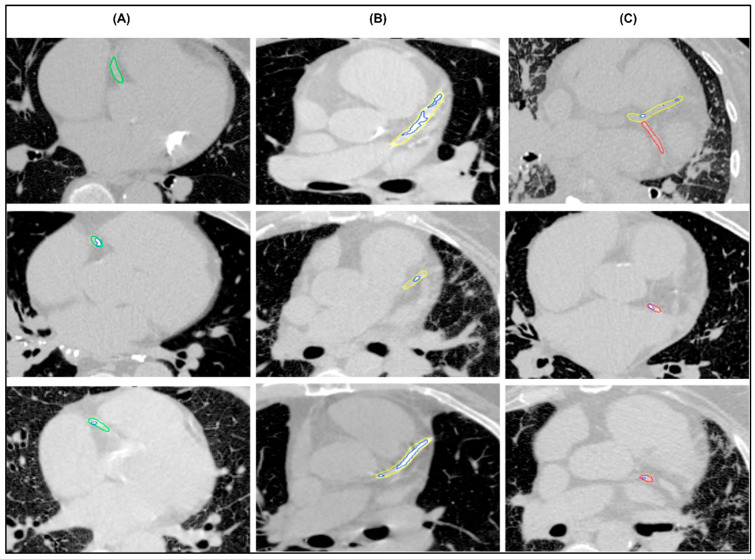
Examples of successful segmentations of (**A**) right coronary artery (green), (**B**) mainstem and left anterior descending artery (yellow), (**C**) left circumflex coronary artery (red) with multiple calcifications (blue).

The proposed model was able to segment the coronary arteries and calcifications among the patients (Figure 3). Inter-observer agreement for the evaluation of AI-derived segmentations was assessed using free-marginal κ analysis, which measures the agreement between two raters, with values closer to 1 indicating stronger agreement. Visual assessment demonstrated high correlation among the two raters, with the majority of segmentations classified as excellent in both assessments (78.5%). Moderate to strong agreement was found for coronary artery segmentation, with κ values of 0.81 (95% CI 0.72–0.90) in the LAD, 0.76 (95% CI 0.66–0.86) in the RCA, and 0.68 (95% CI 0.57–0.79) in the LCX. Moderate to strong agreement was also found for calcification segmentation with κ values of 0.85 (95% CI 0.77–0.94) in the LAD, 0.85 (95% CI 0.77–0.94) in the RCA and 0.79 (95% CI 0.69–0.88) in the LCX. The full assessment results are summarised in Table 2.

### 3.3. Stage 3: Accuracy of Calcification Detection

#### 3.3.1. Accuracy of Binary Detection

The performance of the DL model for coronary artery calcification binary detection was evaluated against the radiologist’s reports. Strong to almost perfect agreement was found for the detection of calcification, with κ values of 0.93 (95% CI 0.90–0.97, *p* < 0.001) for LAD, 0.82 (95% CI 0.77–0.87, *p* < 0.001) for RCA and 0.82 (95% CI 0.77–0.87, *p* < 0.001) for LCX. For the presence of calcification in any coronary artery, the κ value was 0.90 (95% CI 0.85–0.94, *p* < 0.001). For the RCA, the model achieved a sensitivity of 88.3%, a specificity 93.6%, a PPV 93.4%, an NPV 88.7%, and an AUC of 0.91. For the mainstem and LAD, sensitivity was 97.3%, specificity 96.5%, PPV 98.5%, NPV 93.9%, and AUC 0.97. The LCX showed sensitivity of 88.2%, specificity 93.5%, PPV 91.7%, NPV 90.7%, and AUC 0.91. Considering the overall presence of calcifications in any segmented coronary artery, the model achieved a sensitivity of 95.1%, a specificity 97.6%, a PPV of 99.1%, an NPV of 87.9%, and an AUC of 0.96 (Table 3).

#### 3.3.2. Association Between DL-Derived Calcification Volume and Disease Severity

The performance of the DL model in quantifying coronary artery calcification was compared with radiologist visual grading. The DL-derived volumetric measurements of calcifications were able to predict expert radiologist severity grading across all segmented coronary arteries (Table 4 and Supplemental Figure S1). For the RCA, DL-derived mean volumes increased with radiologist grading: 0.028 ± 0.050 mL for mild, 0.107 ± 0.089 mL for moderate, and 0.309 ± 0.049 mL for severe cases. Similarly, for the mainstem and LAD, mean volumes were 0.040 ± 0.088 mL, 0.155 ± 0.090 mL, and 0.387 ± 0.090 mL for mild, moderate, and severe grades, respectively. For the LCX, DL volumes were 0.013 ± 0.090 mL for mild, 0.040 ± 0.034 mL for moderate, and 0.116 ± 0.021 mL for severe calcifications. The findings indicate that higher calcification volumes are strongly associated with higher severity scoring by radiologists. The regression coefficient B was 28.31 (Wald = 154.54, *p* < 0.001) for the RCA, 28.65 (Wald = 196.26, *p* < 0.001) for the LAD and 77.52 (Wald = 145.03, *p* < 0.001) for the LCX.

## 4. Discussion

CAD remains one of the leading causes of mortality. Expediting its detection and diagnosis is therefore important to reducing associated morbidity and mortality. This paper presents an AI tool for the purpose of segmenting and detecting calcifications of the RCA, LAD and LCX on non-ECG-gated non-contrast CT scans. The proposed deep learning model was trained and evaluated using retrospective contrast-enhanced and non-contrast CT scans from patients undergoing investigation for PH. Expert visual assessment of the model outputs showed moderate to strong agreement for both coronary artery segmentation and calcification detection. For binary detection of calcification, the model demonstrated high sensitivity and specificity values and strong to almost perfect agreement with expert opinion.

Conventionally, the detection and quantification of coronary calcification have been performed using ECG-gated CT scans to minimise motion artefacts and improve their diagnostic accuracy [7]. However, several previous studies have highlighted the comparability of diagnostic accuracy for calcification detection in both ECG-gated and non-ECG-gated CT scans [24,25]. Non-contrast CT scans of the thorax are widely performed without ECG gating for a range of indications [26]. Although calcification serves as an obvious landmark of CAD, and its reporting has been emphasised by both the SCCT and STR, its presence and severity in non-dedicated CT scans remain infrequently reported in real clinical practice [11,27]. This work demonstrates that AI can accurately detect and quantify coronary artery calcifications on such scans, indicating the potential for opportunistic screening for CAD in patients undergoing investigations for other pathologies. Our results are consistent with previous studies and additionally support the potential of DL models in achieving high-quality segmentation and calcification detection [15,16,17].

Unlike prior studies that have focused primarily on total calcium scoring or general stenosis prediction across all coronary arteries [8,9,10,15,16,17], our approach targeted artery-specific calcification detection, offering greater alignment with clinical reporting and international societies’ recommendations. In the proposed model, the manual annotations included four categories: RCA, mainstem and LAD, LCX, and a single category for calcifications. To enhance anatomical specificity, mask-based logical operations were used to assign each calcification voxel to its corresponding artery, producing anatomically distinct labels. This refined labelling enabled the development of a more anatomically meaningful segmentation model. Moreover, final predictions were obtained by ensembling the five cross-validated models. While this increases computational overhead at inference time, it substantially improves prediction robustness and accuracy. Importantly, because the model operates only on the cropped heart region, inference remains fast and practical, even on standard hardware. Hence, our model performed automated coronary artery segmentation and detection of calcifications for the RCA, LAD and LCX with high accuracy. This could be particularly advantageous for the detection of incidental coronary artery calcifications, which may otherwise be overlooked, and could facilitate early identification of CAD in patients undergoing CT for other suspected disorders [11,26,27,28].

Importantly, the proposed model not only detects the presence of coronary artery calcifications but is also able to yield clinically meaningful measurements of calcification volume. The DL-derived calcification volumes were found to predict severity as graded by expert cardiothoracic radiologists. Interestingly, a larger regression coefficient was found for the LCX compared to the RCA and LAD, indicating a considerably stronger influence of LCX on the outcome compared to the other variables. Among the three coronary arteries, the effects observed were statistically significant, as indicated by high Wald statistics, confirming the associations are unlikely due to random chance. In addition, the strong relationship between DL-derived volumes and the reference standard was supported by highly significant *p*-values, highlighting the validity and reliability of the model’s predictive performance [29,30,31].

Recent studies have demonstrated the feasibility of automated coronary artery calcium detection and quantification using DL approaches on non-contrast CT. A study evaluated a multi-task DL model in a large multicentre cohort, reporting a sensitivity of 73% and specificity of 98%, with a Cohen’s κ of 0.81 [16]. Another study assessed automated CAC scoring on routine chest CT, demonstrating excellent agreement with reference standards (ICC = 0.94) [32]. Compared with the previous studies, the proposed model achieved high diagnostic performance, with overall sensitivity of 95%, specificity of 98%, PPV of 99%, NPV of 88% and an AUC of 0.96 for overall calcification detection. At the vessel level, Cohen’s κ ranged from 0.82 to 0.93, indicating strong agreement with the reference standard. These findings demonstrate that robust vessel-specific segmentation and volume quantification of coronary calcifications can be achieved on non-ECG-gated, non-contrast CT scans.

The findings of this study have practical implications and are consistent with previous studies highlighting the value of automated coronary artery analysis [12,15,16,17,32]. Manual segmentation and assessment of coronary arteries is inherently time-consuming and subjective. This process is susceptible to variability and errors, particularly given its repetitive and technically demanding nature [12]. As the diagnosis and risk stratification of CAD rely on CT imaging, the development of a high-quality, fully automated segmentation approach is of clear clinical relevance [15,16,17]. The proposed model has the potential to reduce reporting time, minimise interobserver variability, and decrease human error, thereby improving efficiency and reproducibility in routine clinical practice. Furthermore, it may assist in identifying patients with a high calcium burden, potentially reducing unnecessary additional imaging or further diagnostic investigations. By streamlining workflow and supporting more consistent interpretation, such automation may also contribute to reducing healthcare costs while enhancing clinical decision-making [15].

A strength of this study is the use of a well-curated retrospective dataset of CT scans with the presence of coronary artery calcifications from patients undergoing investigation for unrelated pathology. Even though only CT scans with at least 100 slices were included in order to maintain segmentation quality, the model failed to segment the coronary arteries in some cases. This was largely due to the inherent difficulty in visually identifying the arteries, even for experienced radiologists, due to poor image quality or cardiac motion artefacts. Such cases were intentionally retained in the dataset to reflect routine clinical practice and to assess model robustness under challenging but realistic conditions. Visual assessment confirmed this challenge, and a high correlation was observed between AI segmentation and the reference standard. Furthermore, the ASPIRE registry does not specifically record information on prior coronary interventions, such as stents or coronary artery bypass grafts. Consequently, our dataset reflects routine non-contrast CT scans, including any artefacts, rather than a curated set of idealised scans, which enhances the generalisability and clinical relevance of our findings. Although metallic implants may produce artefacts that could potentially affect calcifications detection, the high diagnostic accuracy observed in our results suggests that their impact on the calcifications detection in this cohort was minimal.

Despite the strengths of this study, there are several limitations that should be considered. Region-based or surface distance metrics are commonly used to evaluate segmentation performance, but were not feasible due to the small size of the coronary arteries and their calcifications. A limitation of this study is the relatively small number of manually annotated scans used for model development, and the fact that the sample size was not determined by a priori power analysis, which reflects the time-intensive nature of expert labelling for coronary calcifications on non-contrast CT. However, the model was evaluated on 100 scans for visual assessment and 473 scans for diagnostic accuracy, demonstrating strong performance across multiple metrics. Although class imbalance is inherent in this cohort, the inclusion of all available routine scans, including challenging cases with artefacts, reflects real-world clinical conditions and supports the generalisability of the findings. The calcification severity was assessed by only two experts, which might introduce a potential subjectivity. Future work will involve testing model performance using CT scans acquired from other institutions using different scanners to ensure generalisability and assessing the diagnostic accuracy on contrast-enhanced large cohorts.

## 5. Conclusions

A deep learning model was able to segment the coronary arteries and detect calcification on non-ECG-gated non-contrast CT scans with high performance. In particular, the model demonstrated sensitivity and specificity values in excess of 95% for the detection of calcifications in the RCA, LAD and LCX, and the measurements of calcification volume derived from the model were found to predict the severity of coronary artery disease as graded by expert cardiothoracic radiologists. The model has the potential to enable opportunistic screening for coronary artery disease on thoracic CT studies performed for other indications, but further external testing is required to ensure its generalisability prior to clinical use.

## Figures and Tables

**Table 1:** Demographic information of the patient population.

		Stage 1 Model Development Contrast (*n* = 27) Non-Contrast (*n* = 42)	Stage 2 Visual Assessment Non-Contrast Cohort (*n* = 100)	Stage 3 Diagnostic Accuracy Non-Contrast Cohort (*n* = 473)
Gender	Male	36	33	175
	Female	33	67	298
Age (years ± SD)		69 ± 13	66 ± 12	64 ± 13
Scan dates		2009–2018	2007–2018	2006–2019
Scan institution		Internal Cohort	Internal Cohort	Internal Cohort (*n* = 376)
				External Cohort (*n* = 97)
Scanner manufacturer	GE LightSpeed Pro 32	3	39	193
	GE LightSpeed VCT	15	52	160
	GE Optima CT660	0	0	2
	SIEMENS SOMATOM Definition	4	2	25
	Canon Aquilion One	47	7	93
CT image slices (mean ± SD)		412 ± 210	250 ± 72	261 ± 69
Ethnicity	White	56	70	377
	Non-White	5	8	37
	Not stated	8	22	59
Diagnosis	Group 1: PAH	8	33	159
	Group 2: PH-LHD	4	17	72
	Group 3: PH-Lung disease	21	14	89
	Group 4: CTEPH	3	7	38
	Group 5: PH unclear/multifactorial	0	0	6
	No PH	6	29	109
	Suspected PE	27	NA	NA
Coronary calcification	RCA	37	51	239
	Mainstem + LAD	58	75	329
	LCX	34	50	212

Pulmonary Arterial Hypertension (PAH), Pulmonary Hypertension (PH), Left Heart Disease (LHD), Chronic Thromboembolic Pulmonary Hypertension (CTEPH), Pulmonary Embolism (PE), Not applicable (NA), Right Coronary Artery (RCA), Left Anterior Descending Artery (LAD), Left Circumflex Artery (LCX).

**Table 2:** The visual assessment of the coronary artery and calcifications segmentation (*n* = 100) across two experienced reporters.

	Reporter 1 (AJS) Scoring 100 Scans				Reporter 2 (TNA) Scoring 100 Scans			
	Excellent	Minor Error	Major Error	NA	Excellent	Minor Error	Major Error	NA
RCA	71	26	3	0	77	21	2	0
Mainstem + LAD	82	17	1	0	94	5	1	0
LCX	56	39	4	1	64	26	9	1
RCA Calcification	39	10	0	51	43	4	2	51
Mainstem + LAD Calcification	63	12	0	25	74	1	0	25
LCX Calcification	37	10	0	53	36	7	4	53
Kappa (95% CI)	RCA				0.76 [0.66–0.86]			
	Mainstem + LAD				0.81 [0.72–0.90]			
	LCX				0.68 [0.57–0.79]			
	RCA Calcification				0.85 [0.77–0.94]			
	Mainstem + LAD Calcification				0.85 [0.77–0.94]			
	LCX Calcification				0.79 [0.69–0.88]			

Right Coronary Artery (RCA), Left Anterior Descending Artery (LAD), Left Circumflex Artery (LCX), Not applicable (NA), Confidence Interval (CI).

**Table 3:** Binary comparison between DL and reference standard for coronary artery calcification detection (presence vs. absence) (*n* = 473).

Calcifications	DL Model	* Reference Standard	Cohen’s Kappa [95% CI]	*p*-Value	Sensitivity	Specificity	PPV	NPV	AUC
RCA	226	239	0.82 [0.77–0.87]	<0.001	88.3%	93.6%	93.4%	88.7%	0.91
Mainstem + LAD	325	329	0.93 [0.90–0.97]	<0.001	97.3%	96.5%	98.5%	93.9%	0.97
LCX	204	212	0.82 [0.77–0.87]	<0.001	88.2%	93.5%	91.7%	90.7%	0.91
Overall presence **	333	347	0.90 [0.85–0.94]	<0.001	95.1%	97.6%	99.1%	87.9%	0.96

Right Coronary Artery (RCA), Left Anterior Descending Artery (LAD), Left Circumflex Artery (LCX), Confidence Interval (CI), Positive Predictive Value (PPV) and Negative Predictive Value (NPV). * Calcifications reported by cardiothoracic radiologists. ** Calcification present in any segmented coronary artery.

**Table 4:** Comparison between DL and reference standard for coronary artery calcification severity (volumes vs. reference standard) (*n* = 473).

Calcifications	Radiologist Grading	DL Volumes in mL (Mean ± STD)	Regression Analysis			
			Coefficient (β)	95% CI	Wald	*p*-Value
RCA	Mild	0.028 ± 0.050	28.31	23.85–32.77	154.54	<0.001
	Moderate	0.107 ± 0.089				
	Severe	0.309 ± 0.049				
Mainstem + LAD	Mild	0.040 ± 0.088	28.65	24.65–32.66	196.26	<0.001
	Moderate	0.155 ± 0.090				
	Severe	0.387 ± 0.090				
LCX	Mild	0.013 ± 0.090	77.52	64.90–90.13	145.03	<0.001
	Moderate	0.040 ± 0.034				
	Severe	0.116 ± 0.021				

Right Coronary Artery (RCA), Left Anterior Descending Artery (LAD), Left Circumflex Artery (LCX), Confidence Interval (CI).

## Data Availability

De-identified data within the ASPIRE registry can be made available for analysis to researchers who provide a methodologically sound proposal that fits within the aims of the ASPIRE registry. The ASPIRE data management committee will assess each proposal and decide within 3 months after submission. A data access agreement signed by the lead researcher and a data sharing agreement between Sheffield Teaching Hospitals and the recipient institution will be required before data is shared. Information and application forms can be found at https://bit.ly/aspire-registry (accessed on 15 January 2026). The trained model can be made available upon reasonable request for research purposes by non-commercial entities. Requests should be directed to the corresponding author.

## Supplementary Materials

The following supporting information can be downloaded at: https://www.mdpi.com/article/10.3390/diagnostics16050646/s1. Figure S1: Examples of the reference standard of calcification scoring. Right coronary artery (green), left anterior descending artery (yellow), left circumflex coronary artery (red) and multiple calcifications (blue). Table S1: The model architecture. The network uses an encoder–decoder design with skip connections (Cat) and operates on 112 × 128 × 160 input patches to produce six output channels. Table S2: Ablation study for binary comparison between DL and reference standard for coronary artery calcification detection. Table S3: The visual assessment scoring criteria.

## Abbreviations

The following abbreviations are used in this manuscript:

ACR	American College of Radiology
AI	Artificial Intelligence
ASPIRE	Assessing the Spectrum of Pulmonary Hypertension Identified at a Referral Centre
AUC	Area under the receiver operating characteristic curve
CAC	Coronary Artery Calcium
CAD	Coronary Artery Disease
CI	Confidence Interval
CNN	Convolutional Neural Network
CT	Computed Tomography
CTEPH	Chronic Thromboembolic Pulmonary Hypertension
DL	Deep Learning
DSC	Dice Similarity Coefficient
ECG	Electrocardiograph
Hu	Hounsfield Unit
LAD	Left Anterior Descending artery
LCX	Left Circumflex artery
NA	Not Applicable
NPV	Negative Predictive Value
PAH	Pulmonary Arterial Hypertension
PE	Pulmonary Embolism
PH	Pulmonary Hypertension
PPV	Positive Predictive Value
RCA	Right Coronary Artery
RHC	Right Heart Catheterisation
SCCT	Society of Cardiovascular Computed Tomography
STR	Society of Thoracic Radiology
