# Supplementary material for: A Novel Fully Automated Deep Learning Model for Coronary Artery Calcification Detection on Computed Tomography
Source: Diagnostics (Basel). 2026 Feb 24;16(5):646. doi: 10.3390/diagnostics16050646 (PMC12984781; doi:10.3390/diagnostics16050646)

Supplementary Materials

Supplemental Tables

Table S1. The model architecture. The network uses an encoder-decoder design with skip connections (Cat) and operates on 112×128×160 input patches to produce six output channels.

Stage	Resolution	Channels	Operation
**Input**	112×128×160	1	-
**Encoder Level 1**	112×128×160	32	Conv×2
**Encoder Level 2**	56×64×80	64	Conv×2, Pool (2×2×2)
**Encoder Level 3**	28×32×40	128	Conv×2, Pool (2×2×2)
**Encoder Level 4**	14×16×20	256	Conv×2, Pool (2×2×2)
**Encoder Level 5**	7×8×10	320	Conv×2, Pool (1×2×2)
**Bottleneck**	7×4×5	320	Conv×2
**Decoder Level 1**	7×8×10	640	Upsample (1×2×2), **Cat(Enc5)**, Conv×2
**Decoder Level 2**	14×16×20	512	Upsample (2×2×2), **Cat(Enc4)**, Conv×2
**Decoder Level 3**	28×32×40	256	Upsample (2×2×2), **Cat(Enc3)**, Conv×2
**Decoder Level 4**	56×64×80	128	Upsample (2×2×2), **Cat(Enc2)**, Conv×2
**Decoder Level 5**	112×128×160	64	Upsample (2×2×2), **Cat(Enc1)**, Conv×2
**Output**	112×128×160	6	1×1×1 Conv

Table S2. Ablation study for binary comparison between DL and reference standard for coronary artery calcification detection.

	Calcifications	Sensitivity	Specificity	PPV	NPV	AUC	Cohen's Kappa [95% CI]
**Model (i) ***	**RCA**	88%	94%	94%	89%	0.91	0.82 [0.77 - 0.87]
	**Mainstem + LAD**	97%	97%	99%	94%	0.97	0.93 [0.90 - 0.97]
	**LCX**	88%	94%	92%	91%	0.91	0.82 [0.77 - 0.87]
	**Overall presence****	95%	98%	99%	88%	0.96	0.90 [0.85 - 0.94]
**Model (ii)**	**RCA**	91%	89%	89%	90%	0.89	0.80 [0.74 - 0.85]
	**Mainstem + LAD**	97%	90%	96%	94%	0.94	0.89 [0.84 - 0.93]
	**LCX**	90%	93%	91%	91%	0.91	0.82 [0.77 - 0.87]
	**Overall presence****	98%	88%	95%	95%	0.93	0.88 [0.83 - 0.93]
**Model (iii)**	**RCA**	84%	90%	90%	85%	0.87	0.75 [0.69 - 0.81]
	**Mainstem + LAD**	97%	97%	98%	94%	0.97	0.93 [0.90 - 0.97]
	**LCX**	82%	92%	89%	86%	0.87	0.74 [0.68 - 0.80]
	**Overall presence****	96%	96%	98%	93%	0.96	0.92 [0.88 - 0.95]

Right Coronary Artery (RCA), Left Anterior Descending Artery (LAD), Left Circumflex Artery (LCX), Confidence Interval (CI), Positive Predictive Value (PPV) and Negative Predictive Value (NPV).

*Proposed model presented in the main manuscript.

**Calcification present in any segmented coronary artery.

Table S3. The visual assessment scoring criteria.

Scale	Description
**1: Excellent segmentation**	Highly reliable segmentation.
**2: Minor error**	Error in segmentation but considered by the observer to not affect the measurements.
**3: Significant error**	Error in segmentation considered by the observer to affect the measurements.

Supplemental Figures

Figure S1. Examples of the reference standard of calcification scoring. Right coronary artery (green), left anterior descending artery (yellow), left circumflex coronary artery (red) and multiple calcifications (blue).

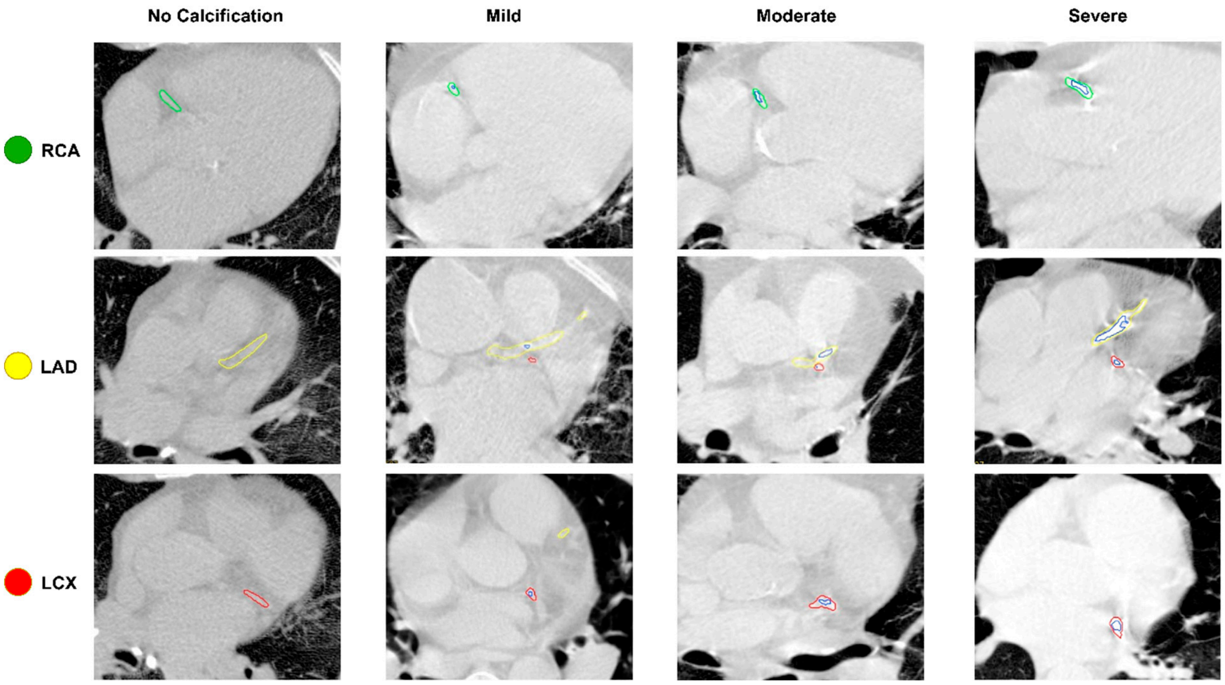

Supplement: Supplementary file 1 [file diagnostics-16-00646-s001.zip › diagnostics-4124792-supplementary.pdf]
